# Factors Affecting V̇O_2_ and Fat Oxidation Responses During Step Incremental Exercise

**DOI:** 10.1111/sms.70110

**Published:** 2025-08-01

**Authors:** Loïs Mougin, Stephen J. Bailey, Mark Burnley, Rhona Pearce, Stephen A. Mears, Michele Zanini

**Affiliations:** ^1^ School of Sport, Exercise and Health Sciences Loughborough University Loughborough UK; ^2^ Loughborough Sport Loughborough University Loughborough UK; ^3^ School of Education, Childhood, Youth and Sport The Open University Milton Keynes UK

**Keywords:** exercise testing, gas exchange, oxygen uptake, physiological assessment, submaximal test, substrate oxidation

## Abstract

Step incremental exercise tests are widely used to assess endurance performance determinants; however, to what extent step duration influences oxygen uptake (V̇O_2_) and carbon dioxide production (V̇CO_2_) is unclear. This study assessed the influence of sampling duration on V̇O_2_, V̇CO_2_, and fat oxidation (Fat_ox_) across different exercise intensities and modalities, and participant age and biological sex during graded exercise tests. A total of 169 participants (41 females; peak V̇O_2_ [V̇O_2_peak]: 54.7 ± 8.6 mL/kg/min) completed a running (*n* = 96) or cycling (*n* = 73) submaximal step test with V̇O_2_ and V̇CO_2_ measured continuously. Each stage was 6 min, and three 30 s averaging windows (2.5–3, 3.5–4, and 4.5–5 min) were compared to the 5.5–6 min reference. Linear mixed models assessed the effects of averaging window, exercise intensity domain, biological sex, age, fitness status, and exercise modality. The analysis included 1031 valid stages (588 running; 443 cycling). In running, V̇O_2_ differences from the 6‐min averaging window were observed at 3 (−28 mL/min), 4 (−15 mL/min), and 5‐min averaging windows (−8 mL/min) (all *p* < 0.001), with similar results for V̇CO_2_. No differences in V̇O_2_ or Fat_ox_ between averaging windows were observed in cycling. These findings were consistent across exercise intensity, fitness status, biological sex, and age. Averaging window differences were < 2% for all conditions. Despite subtle differences, 3‐, 4‐, and 5‐min averaging windows do not meaningfully impact V̇O_2_, V̇CO_2_, or Fat_ox_ compared to a 6‐min stage duration. In conclusion, stage durations of ≥ 3 min may be sufficient to estimate these variables in running and cycling in participants with a wide range of characteristics.

## 1 Introduction

Maximal oxygen uptake (V̇O_2_max), exercise economy/efficiency, and fractional utilisation at lactate thresholds are key physiological determinants of endurance performance [1] as they collectively reflect the capacity for oxygen utilization and translation to speed or power output for a given racing distance. Recent advancements in devices for measuring pulmonary gas exchange have enhanced the practicality of assessing these physiological determinants in a wider range of settings, with well‐established and validated exercise testing protocols used to determine peak oxygen uptake, exercise economy/efficiency, and lactate thresholds [2]. These exercise tests typically comprise a series of stepwise increments in power output or locomotory velocity, each lasting several minutes, until task failure or until a desired exercise intensity is achieved. These testing protocols also permit the measurement of substrate oxidation via indirect calorimetry, potentially aiding optimization of athlete nutrition and performance [3, 4]. While these metabolic responses are useful for athletes, coaches, and practitioners, it is challenging to assess all these parameters in a single test owing to concerns regarding differences in the optimal stage duration required for accurate measurement of all the responses [2]. From a practical perspective, protocols assessing multiple parameters (e.g., V̇O_2_max, economy, and lactate thresholds) in a single visit are advantageous by reducing day‐to‐day variability in potential confounding factors such as nutritional status, exercise, and equipment reliability, which can compromise inter‐test comparisons [5]. Moreover, carrying out the assessments in a single session reduces the logistical and physiological load on the athletes, facilitating the integration of a physiological assessment into the training and racing schedule of the athletes.

For determination of metabolic thresholds, stage durations of 3–6 min have been recommended to achieve a steady‐state blood lactate (BLa) concentration [2], or to capture characteristic lactate kinetics that enable the detection of changes between stages at higher intensities, while shorter test durations are preferred for V̇O_2_max determination as stages ≥ 4 min may result in lower V̇O_2_max estimates [6, 7]. Early experimental comparisons demonstrated no meaningful differences in key physiological variables between 3‐ and 10‐min stage durations, providing a foundational rationale for adopting shorter stages in step tests [8]. For the evaluation of exercise economy, calculated from oxygen and carbon dioxide uptake (V̇O_2_ and V̇CO_2_), 3–8 min stages have been shown to elicit a reliable steady‐state V̇O_2_ in trained athletes [9, 10, 11, 12, 13], while a duration ≥ 4 min has been recommended for running economy assessment across populations [14]. Selecting an optimal stage duration for accurate determination of maximal fat oxidation (Fat_ox_) is more challenging, as longer stage durations (up to 10 min) have been suggested to be required to reach a steady‐state in substrate oxidation [15]. However, longer stage durations may also necessitate the use of larger increments between stages to limit the total exercise duration, which can compromise the accuracy of substrate oxidation estimates [5], or overestimate fat contribution to energy turnover [16]. Though evidence from separate studies suggests that a stage duration of 4 to 5 min is likely to elicit a metabolic steady‐state [2], the effect of stage duration in athletes has not been investigated yet.

In addition to stage duration, the accuracy of V̇O_2_ and Fat_ox_ estimates during stepwise incremental exercise will be influenced by exercise intensity [17]. Indeed, V̇O_2_ kinetics differ between the moderate, heavy, and severe exercise intensity domains, with the development of a V̇O_2_ slow component during heavy intensity exercise delaying the attainment of a steady‐state V̇O_2_ (or a continuous rise at severe intensity) compared to moderate intensity exercise [18]. Furthermore, the averaging window used for pulmonary gas exchange analysis is another variable with the potential to influence V̇O_2_ and Fat_ox_ estimations. The mean response over the last minute of each step is typically used for respiratory gases to calculate exercise economy, to minimize the influence of erroneous breath readings during exercise [14]. Averaging windows of 30 s [19] and ≥ 2 min [17] have also been used to measure respiratory gases during incremental tests. It remains unclear whether variations in the analysis averaging window may influence V̇O_2_, V̇CO_2_, and Fat_ox_, particularly across different stage durations.

Differences in optimal stage duration may also depend on exercise modality. For instance, although Achten et al. [17] validated a protocol with a 3 min stage duration to measure Fat_ox_ in cycling [17], the accuracy of this protocol for other exercise modalities, such as running or rowing, is unclear. Cycling protocols typically involve continuous incremental exercise since capillary blood samples for BLa assessment can be made during exercise. However, this is often not feasible in other modalities, such as running, where breaks of 30 s to 1 min are typically required for BLa assessment [8, 20]. Although these breaks are not likely to affect lactate threshold determination [21], their effect on metabolic rate may influence gas exchange kinetics and the timeframe required to attain a steady state [22], and may reduce the carryover effect from previous stages [17], which may collectively alter substrate oxidation measurements. Assessing stage duration effects in different exercise modalities is important as higher Fat_ox_ has been reported in running compared to cycling at moderate intensities [23], but further research is required to more broadly assess responses during stepwise incremental exercise.

Fitness status may also necessitate adjustments in protocol design [24], as populations with lower aerobic capacity (typically estimated from their V̇O_2_max) often require longer stage durations to achieve a steady state due to a slower V̇O_2_ kinetics [25, 26, 27]. Similarly, factors such as age, with older individuals presenting slower V̇O_2_ kinetics [28]; and biological sex, with faster V̇O_2_ kinetics in young males [29] and a greater contribution of the V̇O_2_ slow component in young females [30], may also influence responses depending on the protocol configuration, although no differences have been shown in adults [31]. Further research is therefore required to assess how step incremental test configuration influences metabolic responses during step incremental exercise across age and biological sex.

The aim of this study was to investigate the effect of stage duration on V̇O_2_, V̇CO_2_, and Fat_ox_ measurements during a step incremental test, via examining differences between 3, 4, 5, and 6 min averaging windows using retrospective testing data from a large cohort of individuals, ranging from active adults to elite athletes. Moreover, the study explored whether these effects differ based on exercise modality (cycling vs. running), exercise intensity, participant fitness status, age, and biological sex. We hypothesized that (1) a stage duration of 4 min would be sufficient to elicit steady‐state for V̇O_2_, V̇CO_2_, and Fat_ox_; (2) exercise intensity domain, measurement duration for averaging the pulmonary gas exchange data, biological sex, and fitness status would not influence these variables; and (3) longer stage duration would be required for older individuals to achieve a steady‐state because of slower V̇O_2_ kinetics [28].

## 2 Methods

### 2.1 Participants

Data from 169 participants were used in this retrospective assessment, with 96 individuals (27 females; age: 39 ± 10 years; V̇O_2_peak: 55 ± 8 mL/kg/min) who completed a running test, while 73 participants (14 females; age: 43 ± 12 years; V̇O_2_peak: 54 ± 9 mL/kg/min) performed a cycling test. Twenty‐three participants (9 females) completed both the running and cycling tests. All participants had previously volunteered for laboratory‐based exercise assessments conducted for physiological profiling, during which they provided verbal and written informed consent. As part of the consent process, participants were informed that their anonymised data may be used for future secondary analyses. This study represents a retrospective analysis of an existing dataset, none of which has been published in any prior form. The original data collection protocol received ethical approval from the Loughborough University Ethics Review Sub‐Committee (Project ID G13‐P1).

### 2.2 Experimental Design

Participants performed graded exercise tests to determine substrate utilization, physiological thresholds, and V̇O_2_peak. Each participant presented to the lab for a single visit. One thousand and thirty‐one stages were analyzed (588 for running tests; 443 for cycling tests; Table 1). Participants completed 8 ± 1 stages in cycling and 8 ± 2 stages in running. All comparisons were performed within each stage to limit differences between stages and tests and the influence of external variables such as acute nutritional status and time of day [5]. Participants avoided strenuous exercise the day before testing and refrained from all activity on the test morning. They were also advised to consume their usual pre‐race meal and beverages for consistency and to avoid large meals within three hours of testing. For the sub‐group of athletes undertaking both running and cycling testing, tests were separated by 4 ± 7 [1–30] days, and participants were instructed to replicate diet and exercise in the 24 h preceding the second testing session as adopted prior to the first testing session.

**TABLE 1:** Participants and test characteristics.

Characteristics	Exercise modality	
	Running (*n* = 96)	Cycling (*n* = 73)
***Participants characteristics***		
Sex	69 M; 27 F	59 M; 14 F
Age (years)	39 ± 10 [19–64]	43 ± 12 [20–73]
Body mass (kg)	73.6 ± 10.9 [47.7–99.6]	77.0 ± 12.5 [52.6–112.5]
V̇O_2_peak (mL/kg/min)	55 ± 8 [36–76]	54 ± 9 [27–73]
Speed—Power at LT (km/h—W)	11.9 ± 2.1 [7.5–15.8]	197 ± 45 [95–300]
V̇O_2_ at LT (mL/kg/min)	42 ± 6 [30–56]	36 ± 7 [19–55]
%V̇O_2_peak at LT	76 ± 5 [66–89]	67 ± 7 [56–82]
Heart rate at LT	155 ± 12 [122–182]	135 ± 14 [93–168]
% maximal heart rate at LT	85 ± 5 [76–94]	77 ± 5 [66–87]
Speed—power at LTP (km/h—W)	13.7 ± 2.3 [8.5–18.2]	249 ± 47 [140–340]
V̇O_2_ at LTP (mL/kg/min)	48 ± 7 [33–64]	44 ± 7 [24–61]
%V̇O_2_peak at LTP	87 ± 4 [75–98]	82 ± 6 [71–94]
Heart rate at LTP	171 ± 12 [134–193]	157 ± 12 [118–184]
% maximal heart rate at LTP	94 ± 3 [88–100]	89 ± 4 [76–94]
***Submaximal incremental exercise***		
Start speed—power (km/h—W)	9.6 ± 1.4 [6.0–13.0]	129 ± 29 [60–200]
Stage increment (km/h—W)	0.7 ± 0.1 [0.5–1.0]	18 ± 3 [10–25]
Stages per test (*n*)	8 ± 1 [5–12]	8 ± 1 [6–11]
Stages analyzed (*n*)	588	443
***Exercise intensity domain***		
Stages at moderate (*n*)	239	195
Stages at heavy (*n*)	178	144
***Fitness status***		
**Good or below**		
*n* (sex)	39 (27 M; 12 F)	36 (32 M; 4 F)
V̇O_2_peak (mL/kg/min)	47 ± 5	47 ± 6
Stages per test* (*n*)	7 ± 1	8 ± 1
Stages analyzed (*n*)	217	205
**Excellent**		
*n* (sex)	57 (42 M; 15 F)	37 (27 M; 10 F)
V̇O_2_peak (mL/kg/min)	61 ± 5	62 ± 5
Stages per test* (*n*)	8 ± 1	8 ± 1
Stages analyzed (*n*)	371	238
***Age***		
**< 40 years**		
*n* (sex)	52 (35 M; 17 F)	27 (14 M; 13 F)
V̇O_2_peak (mL/kg/min)	58 ± 8	59 ± 8
Stages per test (*n*)	8 ± 1	8 ± 1
Stages analyzed (*n*)	322	165
**≥ 40 years**		
*n* (sex)	44 (34 M; 10 F)	46 (45 M; 1 F)
V̇O_2_peak (mL/kg/min)	52 ± 8	52 ± 10
Stages per test (*n*)	8 ± 1	8 ± 1
Stages analyzed (*n*)	266	278
***Biological sex***		
**Females**		
*n*	27	14
V̇O_2_peak (mL/kg/min)	51 ± 9	56 ± 9
Stages per test* (*n*)	8 ± 1	8 ± 1
Stages analyzed (*n*)	154	87
**Males**		
*n*	69	59
V̇O_2_peak (mL/kg/min)	57 ± 8	54 ± 10
Stages per test* (*n*)	8 ± 1	8 ± 1
Stages analyzed (*n*)	434	356
***Within‐participant exercise modality***		
*n* (sex)	23 (14 M; 9 F)	
V̇O_2_peak (mL/kg/min)	55 ± 7	53 ± 9
Stages per test (*n*)	8 ± 1	8 ± 1
**Stages analyzed (*n*)**		
Moderate intensity	54	44
Heavy intensity	44	45

*Note:* Mean ± SD [minimum–maximum]. Difference in stages per test between conditions (i.e., fitness status, age, biological sex, within‐participant exercise modality).

* *p* < 0.05 for running.

#### 2.2.1 Cycling Test

**FIGURE 1:**
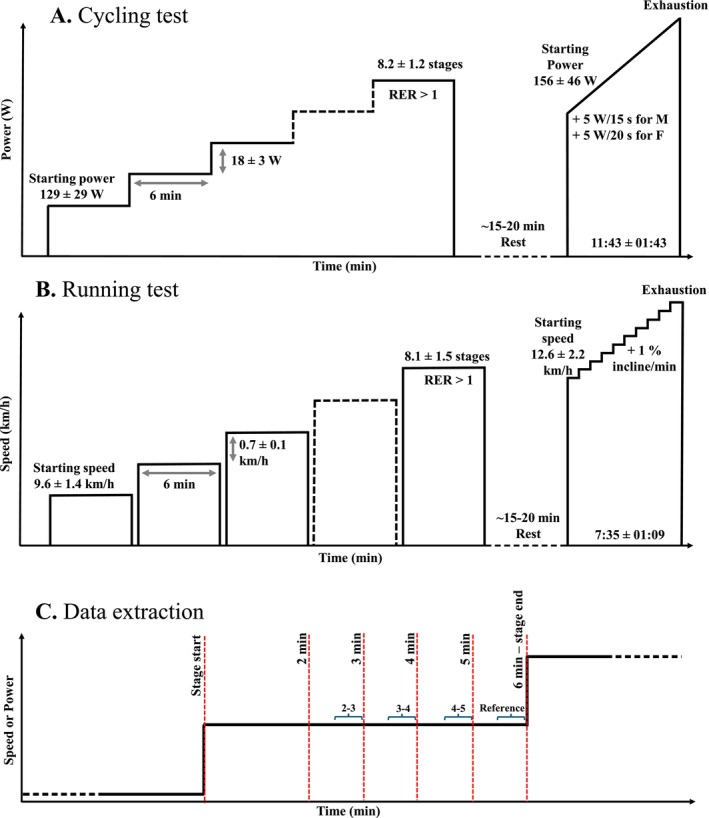
Cycling (A) and running (B) test procedures. Description of data extraction for each stage of incremental step exercise (C).

The cycling submaximal incremental exercise was performed either on a turbo trainer (Kickr Smart Trainer, Wahoo Fitness, Atlanta, USA) using the participant's own bicycle or on an SRM cycle ergometer (Schoberer Rad Messtechnik, Jülich, Germany), with similar power output accuracy demonstrated between the two devices [32]. The test was composed of 6 min stages until a respiratory exchange ratio (RER) > 1.0 was reached. Starting power varied between 60 and 200 W, and the step increment to each stage varied between 10 and 25 W (Figure 1A), with these variables estimated based on the participants' training experience. A capillary blood sample was collected from the earlobe in the last 30 s of each stage without exercise interruptions, and pulmonary gas exchange was measured continuously throughout the test. Following 15–20 min of rest after the end of the incremental test, a ramp test to task failure was performed to determine V̇O_2_peak. The starting power (60–260 W) was determined based on training background, with an increase of 20 W/min for males or 15 W/min for females until task failure, aiming for a test duration of 8–12 min. The highest 1 min rolling average V̇O_2_ of the exercise was used to determine V̇O_2_peak, to reduce breath‐by‐breath variability.

#### 2.2.2 Running Test

The running submaximal incremental exercise was performed on a treadmill (mercury h/p/cosmos, Nussdorf, Germany). The test was composed of 6 min stages, interspaced by 30 s rest for capillary blood sampling, until an RER > 1.0 was reached. Pulmonary gas exchange was measured continuously throughout the test. Starting speed ranged between 6 and 12.5 km/h and increments between 0.5 and 1.0 km/h (Figure 1B), determined based on the participants' training background. The gradient was fixed to 1% for the submaximal stages. Following 15–20 min of rest after completion of the initial incremental test, another incremental test was performed to task failure to assess V̇O_2_peak. The starting speed was set 2 km/h lower than the final stage of the submaximal exercise, with an initial incline of 1% and increasing thereafter by 1% every minute until task failure, aiming for a test duration of 8–12 min. The highest 1 min rolling average V̇O_2_ of the exercise was used to determine V̇O_2_peak, to reduce breath‐by‐breath variability.

### 2.3 Gas Exchange and Blood Lactate Concentration

Throughout the test, participants wore a low‐dead‐space mask and breathed through an impeller turbine assembly (Jaeger Triple V, Jaeger GmbH, Hoechberg, Germany) to measure O_2_ and CO_2_ concentrations in expired air via an open‐circuit metabolic cart (Jaeger Vyntus CPX, Carefusion, San Diego, CA, USA). Inspired and expired‐gas volumes and concentrations were continuously measured on a breath‐by‐breath basis using paramagnetic (O_2_) and infrared (CO_2_) analyzers (Jaeger Vyntus CPX, Carefusion, San Diego, CA, USA) with a 75 ms typical rise time (T10–90), via a capillary line. These analyzers were calibrated before each test using a known gas mixture (16% O_2_ and 5% CO_2_) and ambient air. The turbine volume transducer was calibrated using a 3‐L syringe (Hans Rudolph, KS, USA). The volume and concentration signals were time‐aligned, accounting for the transit delay in capillary gas and analyzer rise time relative to the volume. Fat oxidation rates were assessed using the equation below proposed by Frayn [33]:

$$\begin{array}{c} \mathrm{Fat}\;\text{oxidation}\;\left(g/\min\right)\\ =1.67\times \dot{V}O_{2}\;\left(L/\min\right)–1.67\times \dot{V}{\mathrm{CO}}_{2}\;\left(L/\min\right)–1.92\times 0.01 \end{array}$$

Blood lactate concentrations were analyzed (Biosen C‐Line Glucose and Lactate analyzer, EKF Diagnostics, Cardiff, UK) from 20 μL earlobe capillary samples.

### 2.4 Data Analysis

During the submaximal incremental exercise tests, breath‐by‐breath data from the last 4 min of each stage were analyzed. Four specific 1‐min time intervals (referred to as “averaging window”) were examined within each stage: 2–3 min (3 min), 3–4 min (4 min), 4–5 min (5 min), and 5–6 min (6 min). For each averaging window (i.e., 3, 4, 5, 6 min), data were averaged over the last 30 s, with the 6 min averaging window set as reference for comparison across other averaging windows. V̇O_2_, V̇CO_2_, and Fat_ox_ were analyzed as the difference between the V̇O_2_/V̇CO_2_/Fat_ox_ in the 6 min averaging window and the V̇O_2_/V̇CO_2_/Fat_ox_ of each averaging window (3, 4, or 5 min). To assess the effect of sampling time, each stage was compared by averaging the last 30 s and the last 60 s of the stage. For each participant, the first stage of each exercise was excluded from the analysis to ensure that the possible priming exercise effect on oxygen consumption was consistent across all stages [34, 35].

During the step test, lactate threshold (LT) was defined as the first rise in blood lactate concentrations from baseline (+0.4–0.5 mmol/L), indicating the onset of increased anaerobic metabolism. Lactate turn point (LTP) was defined as a rapid and sustained increase in blood lactate concentrations (typically above +1.5 mMol/L) between two subsequent stages [36]. Exercise intensity domains were defined using LT and LTP, with moderate intensity for stages performed below LT, heavy intensity between LT and LTP, and severe intensity above LTP. Any stage performed at severe intensity was excluded from all analyses, as steady‐state conditions are not achieved at this intensity [18]. Moreover, at severe exercise intensities, increased glycolytic flux produces a high concentration of H^+^ ions, which are buffered by blood bicarbonate (HCO_3_^−^). This buffering reaction generates excess CO_2_ that is exhaled through increased ventilation (hyperpnea) [4]. Consequently, anytime bicarbonate buffering occurs, substrate oxidation cannot be calculated by pulmonary gas exchange. Therefore, only stages within the moderate and heavy intensity domains were included for exercise intensity comparisons (see details in Table 1). To avoid transitional phases and improve the accuracy of intensity domain‐specific interpretations, stages performed at a speed or power corresponding to LT or LTP ±2% were excluded, accounting for variability in metabolic thresholds determination. Participant fitness status was sub‐classified as “Excellent” or “Good (or below)” based on ACSM classification [24] depending on their V̇O_2_peak being above or below 50 mL/min/kg (females) or 55 mL/min/kg (males). Biological sex and age comparisons (with individuals classified as seniors if aged between 18 and 39 years and masters if aged 40 years or older) were also performed. Furthermore, for a direct comparison of exercise modality, a within‐participant analysis was conducted for the athletes that completed both cycling and running tests. The effect of averaging window was assessed between exercise modality for all the stages below LTP as above described.

### 2.5 Statistical Analyses

Shapiro–Wilk tests were initially conducted to check the normality of the dependent variables. No violation of normality was observed and, as such, data are presented as mean ± SD. Statistical analyses were conducted using RStudio (version 2023.03.0, RStudio, PBC, Boston, MA, USA). Linear Mixed Models (LMM) were performed to examine the differences between averaging window, measurement duration, intensity domains, fitness status, age, and biological sex on V̇O_2_, V̇CO_2_, and Fat_ox_ for both running and cycling modalities. Outliers were removed if the difference in V̇O_2_, V̇CO_2_, or Fat_ox_ between a given window and the 6 min reference exceeded 3 standard deviations from the group mean as the datasets were normally distributed (these outliers also exceeded 3 median absolute deviations from the median; [37]). In running, outliers removed for V̇O_2_ were 5 at 3 min, 2 at 4 min, and none at 5 min; while in cycling, 5 were removed at 3 min, 3 at 4 min, and 1 at 5 min. Fixed (averaging window, measurement duration, intensity domain, fitness status, age, biological sex) and random (participants) effects for LMM were fit for each dependent variable. The most appropriate model was chosen using the smallest Hurvich and Tsai's criterion (AICC) in accordance with the principle of parsimony. Main effects were calculated for averaging window, measurement duration, intensity domain, fitness status, age, and biological sex; and interaction effects were evaluated for averaging window × measurement duration/intensity domain/fitness status/age/biological sex. For the participants taking part in both cycling and running testing, the main effect of averaging window modality was calculated to ensure that the sub‐group was representative of the overall cohort of athletes. Similarly to the other conditions, the main effect of exercise modality and averaging window × exercise modality interaction were also analyzed. Pairwise comparisons of estimated marginal means were performed using the Holm‐Bonferroni adjustment to control for multiple comparisons. Effect sizes were calculated as partial‐eta squared (*η*_p_^2^) and quantified as small (0.01–0.06), medium (0.06–0.14), and large (> 0.14). Statistical significance was set at *p* < 0.05. On figures, the clouds of points indicate all data points, the boxplots indicate the data distribution, the median and the 1st quartile (25th percentile) and the 3rd quartile (75th percentile), and the one‐sided violin plots indicate the data distribution.

## 3 Results

### 3.1 Averaging Window

**FIGURE 2:**
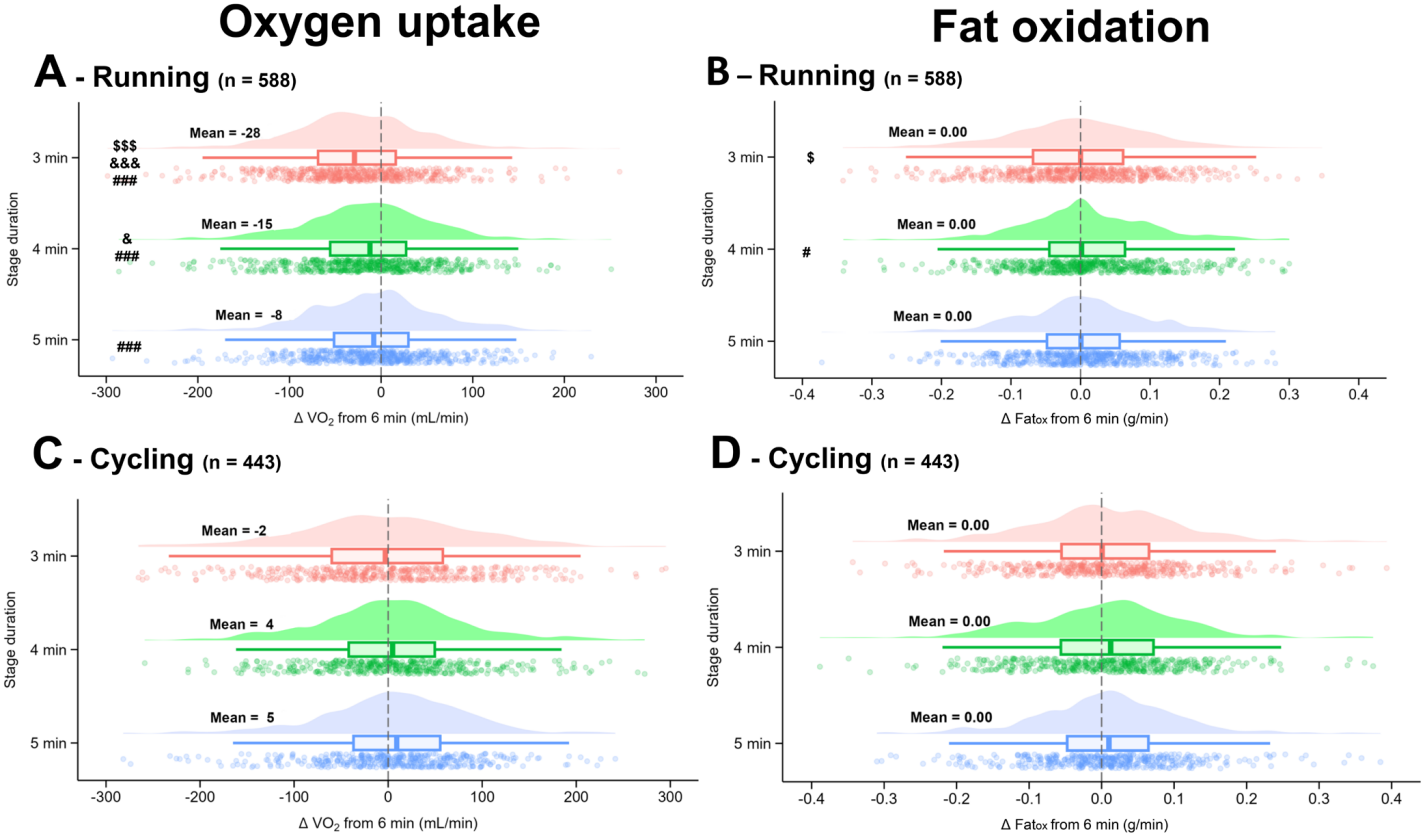
Differences in V̇O_2_ and Fat_ox_ measured after 3, 4, 5 min compared to 6 min for each stage during an incremental step test in running (A and B, respectively) and cycling (C and D, respectively). Data in bold represent the mean for each condition. The clouds of points indicate all data points, the boxplots indicate the data distribution, the median, and the 1st quartile (25th percentile) and the 3rd quartile (75th percentile), and the one‐sided violin plots indicate the data distribution for the averaging window. ^#^Different to 6 min; ^&^different to 5 min; ^$^different to 4 min; 3 symbols for *p* < 0.001; 1 symbol for *p* < 0.05.

For V̇O_2_, a main effect of the averaging window was found for running (*p* < 0.001; *F* = 33.0; *η*_p_^2^ = 0.05; Figure 2A) but not for cycling (*p* = 0.214; *F* = 1.5; *η*_p_^2^ = 0.003; Figure 2C). During running, a lower V̇O_2_ compared to 6 min was found for an averaging window of 3 (−29 mL/min), 4 (−16 mL/min), and 5 min (−9 mL/min; all *p* < 0.001); V̇O_2_ measured at 3 min was lower compared to 4 and 5 min (*p* < 0.001), and at 4 min compared to 5 min (*p* = 0.018).

For Fat_ox_, a main effect of averaging window was found for running (*p* = 0.019; *F* = 3.3; *η*_p_^2^ = 0.01; Figure 2B) but not for cycling (*p* = 0.331; *F* = 1.1; *η*_p_^2^ = 0.003; Figure 2D). During running, compared to 4 min, Fat_ox_ was lower at 6 min (*p* = 0.037) and 3 min (*p* = 0.037).

For V̇CO_2_, a main effect of the averaging window was found for running (*p* < 0.001; *F* = 37.1; *η*_p_^2^ = 0.06; Table S2) and for cycling (*p* < 0.001; *F* = 8.3; *η*_p_^2^ = 0.02; Table S2). During running, a lower V̇CO_2_ compared to 6 min was found for an averaging window of 3 (−34 mL/min), 4 (−12 mL/min; all *p* < 0.001) min, and V̇CO_2_ measured at 3 min was lower compared to 4 and 5 min (*p* < 0.001). During cycling, a higher V̇CO_2_ compared to 6 min was found for an averaging window of 3 (+20 mL/min), 4 (+20 mL/min) and 5 min (+16 mL/min; all *p* < 0.001).

### 3.2 Measurement Duration

In running, when measurement durations of 30 and 60 s were compared, no main effect of measurement duration was observed on V̇O_2_ (*p* = 0.684; *F* = 0.2; *η*_p_^2^ < 0.001), Fat_ox_ (*p* = 0.099; *F* = 2.7; *η*_p_^2^ < 0.001) but lower values were observed on V̇CO_2_ at 60 s (*p* < 0.001; F = 13.7; *η*_p_^2^ = 0.003). An averaging window × measurement duration interaction effect was observed during running for V̇O_2_ (*p* = 0.010; *F* = 3.8; *η*_p_^2^ = 0.003; Table S1) but not for Fat_ox_ (*p* = 0.901; *F* = 0.2; *η*_p_^2^ < 0.001) and V̇CO_2_ (*p* = 0.165; *F* = 1.7; *η*_p_^2^ = 0.001). No significant difference between 30 and 60 s was observed when comparing V̇O_2_ across the 3, 4, 5, and 6 min averaging windows.

During cycling, no main effect of measurement duration was observed for V̇O_2_ (*p* = 0.497; *F* = 0.5; *η*_p_^2^ < 0.001), V̇CO_2_ (*p* = 0.588; *F* = 0.3; *η*_p_^2^ < 0.001), or Fat_ox_ (*p* = 0.282; *F* = 1.2; *η*_p_^2^ < 0.001). Similarly, no averaging window × measurement duration effect was observed on V̇O_2_ (*p* = 0.579; *F* = 0.7; *η*_p_^2^ < 0.001), V̇CO_2_ (*p* = 0.949; *F* = 0.1; *η*_p_^2^ = 0.002) or Fat_ox_ (*p* = 0.198; *F* = 1.6; *η*_p_^2^ < 0.001).

### 3.3 Exercise Intensity Domain

**FIGURE 3:**
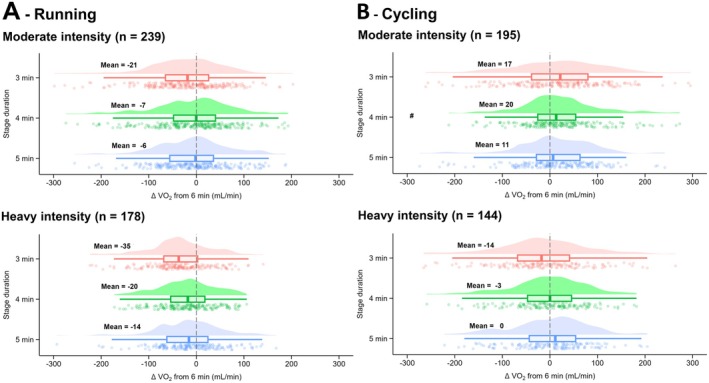
Differences in V̇O_2_ measured after 3, 4, and 5 min compared to 6 min for stages performed in the moderate or heavy intensity domain, in running (A) and cycling (B). Data in bold represent the mean for each condition. The clouds of points indicate all data points, the boxplots indicate the data distribution, the median, and the 1st quartile (25th percentile) and the 3rd quartile (75th percentile), and the one‐sided violin plots indicate the data distribution for the averaging window. ^#^Different from 6 min; 1 symbol for *p* < 0.05.

When moderate and heavy exercise intensity were compared during running for V̇O_2_, no main effect of exercise intensity (*p* = 0.055; *F* = 3.7; *η*_p_^2^ = 0.05) nor averaging window × intensity interaction effect (*p* = 0.224; *F* = 1.5; *η*_p_^2^ = 0.004; Figure 3A) were observed. Similarly, no main effect of exercise intensity (*p* = 0.288; *F* = 1.1; *η*_p_^2^ = 0.003) nor averaging window × intensity interaction effect (*p* = 0.114; *F* = 2.0; *η*_p_^2^ = 0.005; Table S2) was observed for Fat_ox_. For V̇CO_2_ during running, no main effect of exercise intensity (*p* = 0.086; *F* = 3.0; *η*_p_^2^ = 0.01) was observed, but an averaging window × intensity interaction effect (*p* = 0.011; *F* = 3.7; *η*_p_^2^ = 0.01; Table S3) was observed. At moderate intensity, a lower V̇CO_2_ was observed at 3 min compared to 6 min (−18 mL/min; *p* = 0.012). At heavy intensity, a lower V̇CO_2_ was observed at 3 min compared to 4, 5, and 6 min (−39 mL/min for 6 min; all *p* < 0.001) and at 4 min compared to 6 min (−18 mL/min; *p* = 0.049).

For cycling, a main effect of exercise intensity was observed for V̇O_2_ (*p* = 0.002; *F* = 9.6; *η*_p_^2^ = 0.03), with a lower ΔV̇O_2_ at heavy intensity (moderate: 9 ± 107 mL/min vs. heavy: −4 ± 72 mL/min), for V̇CO_2_ (*p* = 0.004; *F* = 8.5; *η*_p_^2^ = 0.03) but not for Fat_ox_ (*p* = 0.864; *F* = 0.03; *η*_p_^2^ < 0.001). An averaging window × intensity interaction effect was observed for V̇O_2_ (*p* = 0.003; *F* = 4.7; *η*_p_^2^ = 0.01; Figure 3B), for V̇CO_2_ (*p* = 0.026; *F* = 3.1; *η*_p_^2^ = 0.01; Table S3), but not for Fat_ox_ (*p* = 0.080; *F* = 2.3; *η*_p_^2^ = 0.01; Table S2). At moderate intensity, a higher V̇O_2_ was observed at 4 min compared to 6 min (*p* = 0.018). Similarly, at moderate intensity, a higher V̇CO_2_ was observed at 3, 4, and 5 min compared to 6 min (all *p* < 0.01).

### 3.4 Fitness Status

**FIGURE 4:**
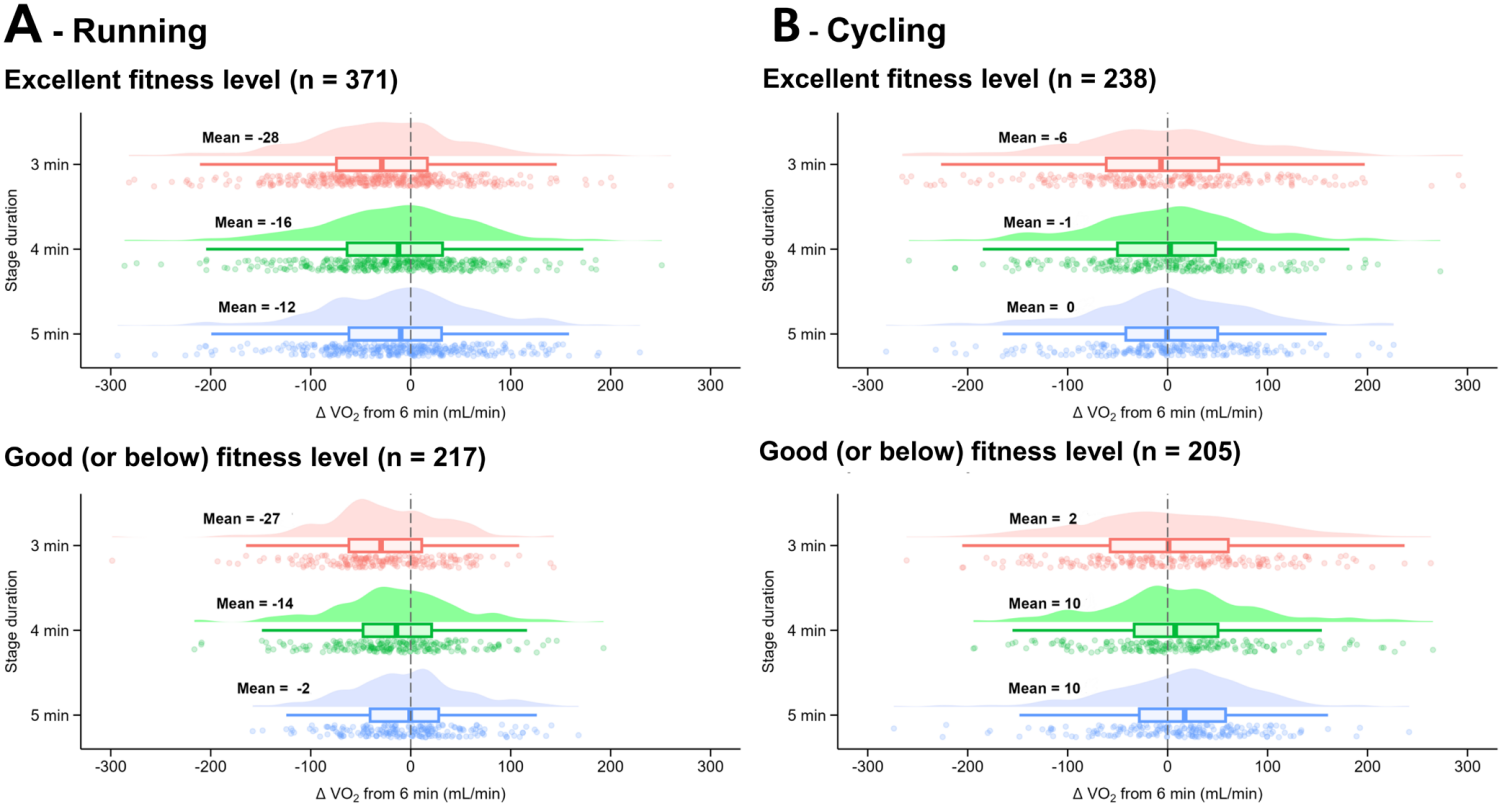
Differences in V̇O_2_ measured after 3, 4, 5 min compared to 6 min for stages performed by individuals with good (or below) or excellent fitness status, in running (A) and cycling (B). Data in bold represent the mean for each condition. The clouds of points indicate all data points, the boxplots indicate the data distribution, the median, and the 1st quartile (25th percentile) and the 3rd quartile (75th percentile), and the one‐sided violin plots indicate the data distribution for the averaging window.

When athletes of different fitness statuses were analyzed, no main effect of fitness status was observed during running for V̇O_2_ (*p* = 0.376; *F* = 0.8; *η*_p_^2^ = 0.002), Fat_ox_ (*p* = 0.195; *F* = 1.7; *η*_p_^2^ = 0.003) or V̇CO_2_ (*p* = 0.323; *F* = 1.0; *η*_p_^2^ = 0.004). No averaging window × fitness status interaction effect was observed during running for V̇O_2_ (*p* = 0.355; *F* = 1.1; *η*_p_^2^ = 0.002; Figure 4A), Fat_ox_ (*p* = 0.641; *F* = 0.6; *η*_p_^2^ < 0.001; Table S2) or V̇CO_2_ (*p* = 0.090; *F* = 2.2; *η*_p_^2^ = 0.004; Table S3).

For cycling, no main effect of fitness status was observed for V̇O_2_ (*p* = 0.107; *F* = 2.6; *η*_p_^2^ = 0.01) or V̇CO_2_ (*p* = 0.074; *F* = 3.2; *η*_p_^2^ = 0.01), but a main effect was observed for Fat_ox_ (*p* = 0.041; *F* = 4.2; *η*_p_^2^ = 0.01). No averaging window × fitness status interaction effect was observed for V̇O_2_ (*p* = 0.460; *F* = 0.9; *η*_p_^2^ = 0.002; Figure 4A) or V̇CO_2_ (*p* = 0.237; *F* = 1.4; *η*_p_^2^ = 0.003; Table S3), but an interaction effect was found for Fat_ox_ (*p* = 0.023; *F* = 3.2; *η*_p_^2^ = 0.01; Table S2), although post hoc analysis did not reveal any difference between averaging window for the two sub‐groups.

### 3.5 Biological Sex

**FIGURE 5:**
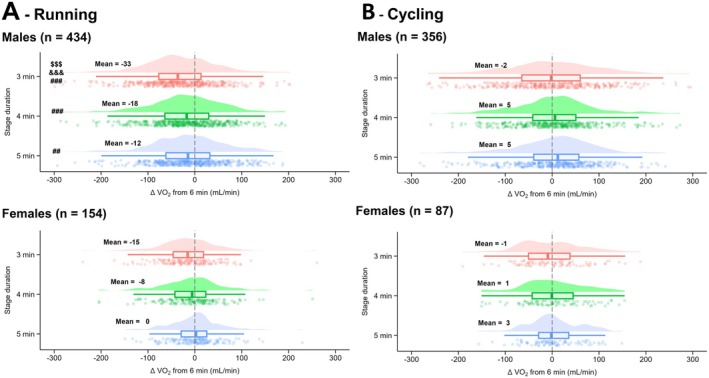
Differences in V̇O_2_ measured after 3, 4, and 5 min compared to 6 min for stages performed by females or males, in running (A) and cycling (B). Data in bold represent the mean for each condition. The clouds of points indicate all data points, the boxplots indicate the data distribution, the median, and the 1st quartile (25th percentile) and the 3rd quartile (75th percentile), and the one‐sided violin plots indicate the data distribution for the averaging window.

When males and females were compared, a main effect of biological sex was observed during running for V̇O_2_ (*p* = 0.011; *F* = 6.5; *η*_p_^2^ = 0.01; greater delta in males). An averaging window × biological sex interaction effect was observed during running for V̇O_2_ (*p* = 0.049; *F* = 2.6; *η*_p_^2^ = 0.004; Figure 5A) with, for males, lower V̇O_2_ at 3 min compared to 4, 5, and 6 min (all *p* < 0.001), and at 4 and 5 min compared to 6 min (*p* < 0.001 and *p* = 0.007, respectively) while no differences were observed between averaging window for females, with the same results observed when expressing V̇O_2_ relative to body mass. No main effect of biological sex was observed during running for Fat_ox_ (*p* = 0.370; *F* = 0.8; *η*_p_^2^ = 0.001) or V̇CO_2_ (*p* = 0.891; *F* = 0.02; *η*_p_^2^ < 0.001). No averaging window × biological sex interaction effect was observed during running for Fat_ox_ (*p* = 0.757; *F* = 0.4; *η*_p_^2^ < 0.001; Table S2) or V̇CO_2_ (*p* = 0.975; *F* = 0.1; *η*_p_^2^ < 0.001; Table S3).

During cycling, no main effect of biological sex was observed for V̇O_2_ (*p* = 0.784; *F* = 0.1; *η*_p_^2^ < 0.001) or V̇CO_2_ (*p* = 0.124; *F* = 2.4; *η*_p_^2^ = 0.01). No averaging window × biological sex interaction effect was observed during cycling for V̇O_2_ (*p* = 0.976; *F* = 0.1; *η*_p_^2^ < 0.001; Figure 5B) or V̇CO_2_ (*p* = 0.343; *F* = 1.1; *η*_p_^2^ = 0.002; Table S3). A main effect of biological sex was observed for Fat_ox_ (*p* = 0.032; *F* = 4.6; *η*_p_^2^ = 0.01; Table S2) but no averaging window × biological sex interaction effect was reported (*p* = 0.152; *F* = 1.8; *η*_p_^2^ = 0.004).

### 3.6 Age

**FIGURE 6:**
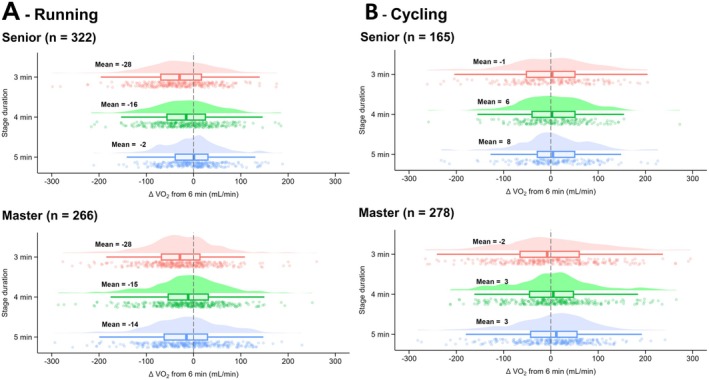
Differences in V̇O_2_ measured after 3, 4, and 5 min compared to 6 min for stages performed by senior (< 40 years) or master (≥ 40 years) individuals, in running (A) and cycling (B). Data in bold represent the mean for each condition. The clouds of points indicate all data points, the boxplots indicate the data distribution, the median, and the 1st quartile (25th percentile) and the 3rd quartile (75th percentile), and the one‐sided violin plots indicate the data distribution for the averaging window.

When seniors and masters participants were compared, during running, no main effect of age was observed for V̇O_2_ (*p* = 0.414; *F* = 0.7; *η*_p_^2^ = 0.001) or V̇CO_2_ (*p* = 0.295; *F* = 1.1; *η*_p_^2^ = 0.002). No averaging window × age interaction effect was observed during running for V̇O_2_ (*p* = 0.091; *F* = 2.2; *η*_p_^2^ = 0.004; Figure 6A) or V̇CO_2_ (*p* = 0.725; *F* = 0.4; *η*_p_^2^ < 0.001; Table S3). A main effect of age was observed for Fat_ox_ (*p* = 0.020; *F* = 5.5; *η*_p_^2^ = 0.01) as well as an averaging window × age interaction effect (*p* = 0.044; *F* = 2.7; *η*_p_^2^ = 0.005), although post hoc analysis did not reveal any difference between averaging windows for the two sub‐groups.

During cycling, no main effect of age was observed for V̇O_2_ (*p* = 0.754; *F* = 0.1; *η*_p_^2^ < 0.001), Fat_ox_ (*p* = 0.670; *F* = 0.2; *η*_p_^2^ < 0.001) or V̇CO_2_ (*p* = 0.372; *F* = 0.8; *η*_p_^2^ = 0.002). No averaging window × age interaction effect was observed during cycling for V̇O_2_ (*p* = 0.924; *F* = 0.2; *η*_p_^2^ < 0.001; Figure 6B), Fat_ox_ (*p* = 0.601; *F* = 0.6; *η*_p_^2^ = 0.001; Table S2) or V̇CO_2_ (*p* = 0.320; *F* = 1.2; *η*_p_^2^ = 0.003; Table S3).

### 3.7 Within‐Participant Exercise Modality

**FIGURE 7:**
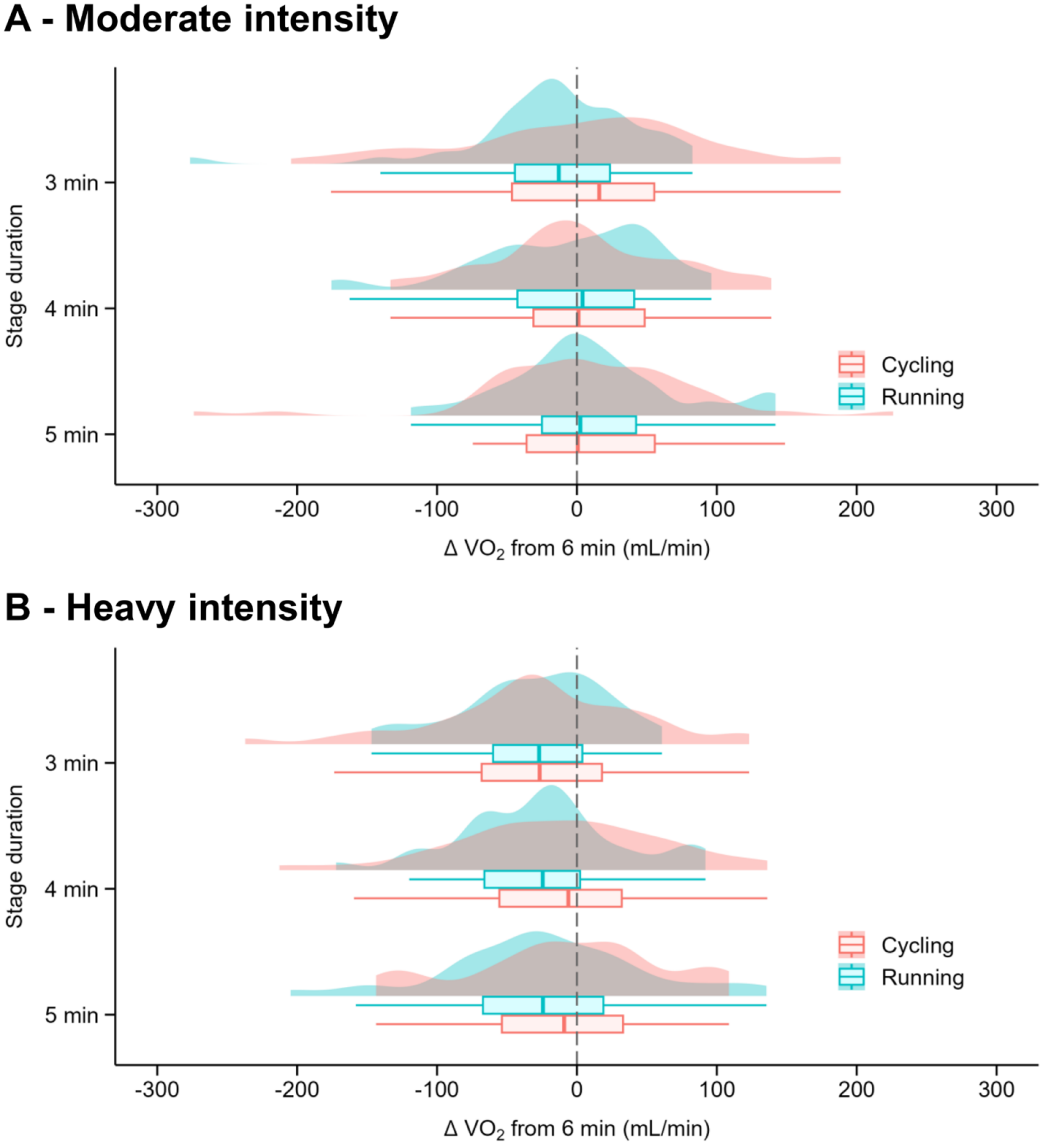
Differences in measuring V̇O_2_ after 3, 4, and 5 min compared to 6 min for stages performed in running (Blue) and cycling (Red) for the 23 participants that undertaken both testing at moderate (A) and heavy (B) intensities. The boxplots indicate the data distribution, the median and the 1st quartile (25th percentile) and the 3rd quartile (75th percentile), and the one‐sided violin plots indicate the data distribution for averaging window and exercise modality. Stages (*n*): moderate intensity: 54 (running), 44 (cycling); heavy intensity: 44 (running); 45 (cycling).

For the sub‐group of participants who completed both running and cycling testing, at moderate intensity, no main effect of exercise modality was observed for V̇O_2_ (*p* = 0.791; *F* = 0.1; *η*_p_^2^ < 0.001; Figure 7), V̇CO_2_ (*p* = 0.130; *F* = 2.3; *η*_p_^2^ = 0.02) or Fat_ox_ (*p* = 0.825; *F* = 0.05; *η*_p_^2^ < 0.001). No main effect of averaging window was observed for V̇O_2_ (*p* = 0.199; *F* = 1.6; *η*_p_^2^ < 0.001; Figure 7), V̇CO_2_ (*p* = 0.125; *F* = 1.9; *η*_p_^2^ = 0.02) or Fat_ox_ (*p* = 0.114; *F* = 2.0; *η*_p_^2^ < 0.001). No averaging window × exercise modality interaction effect was observed on V̇O_2_ (*p* = 0.859; *F* = 0.3; *η*_p_^2^ = 0.002; Figure 7) nor V̇CO_2_ (*p* = 0.408; *F* = 1.0; *η*_p_^2^ = 0.01). An averaging window × exercise modality interaction effect was observed for Fat_ox_ (*p* = 0.047; *F* = 2.7; *η*_p_^2^ = 0.02), although no difference between cycling and running was observed at 3, 4, and 5 min.

At heavy intensity, no main effect of exercise modality was observed for V̇O_2_ (*p* = 0.937; *F* = 0.0; *η*_p_^2^ < 0.001; Figure 7), V̇CO_2_ (*p* = 0.681; *F* = 0.2; *η*_p_^2^ < 0.001) or Fat_ox_ (*p* = 0.146; *F* = 2.2; *η*_p_^2^ = 0.02). No main effect of averaging window was observed for V̇O_2_ (*p* = 0.050; *F* = 2.0; *η*_p_^2^ < 0.001; Figure 7), V̇CO_2_ (*p* = 0.261; *F* = 1.3; *η*_p_^2^ = 0.002) or Fat_ox_ (*p* = 0.336; *F* = 1.1; *η*_p_^2^ = 0.02). No averaging window × exercise modality interaction effect was observed for V̇O_2_ (*p* = 0.400; *F* = 1.0; *η*_p_^2^ = 0.01; Figure 7), V̇CO_2_ (*p* = 0.119; *F* = 2.0; *η*_p_^2^ = 0.02) or Fat_ox_ (*p* = 0.126; *F* = 1.9; *η*_p_^2^ = 0.02).

## 4 Discussion

This study examined the impact of averaging window, as a proxy of stage duration, on V̇O_2_, V̇CO_2_, and Fat_ox_ during step incremental cycling and running protocols in a large cohort of athletes, and assessed differences across several sub‐grouped conditions. Lower V̇O_2_, V̇CO_2_, and Fat_ox_ were observed at 3, 4, and 5 min compared to 6 min in running, although the magnitude of these differences was very small (< 2%) and within the measurement error of these variables. In cycling, no V̇O_2_, V̇CO_2_, or Fat_ox_ differences were found between any averaging window. Fitness status, age, and biological sex also did not influence these findings, suggesting that a similar step test protocol can be applied across a wide range of populations.

### 4.1 Averaging Window

During running, differences in V̇O_2_, V̇CO_2_, and Fat_ox_ were observed between averaging windows of 3, 4, 5, or 6 min, with lower values for shorter averaging windows (e.g., −28 mL/min Δ between 3 and 6 min for V̇O_2_). While the differences in V̇O_2_, V̇CO_2_, and Fat_ox_ between the averaging windows were statistically significant, their practical relevance appears limited due to the effect size magnitude (i.e., < 0.06) and could be partly attributed to the large sample size and statistical power in the current study. For example, the observed −28 mL/min Δ between 3 and 6 min for V̇O_2_ represents only ~1% of the absolute V̇O_2_ required for the exercise. This difference further decreases to ~15 mL/min when comparing the 4 vs. 6 min windows. Similar trends were observed for V̇CO_2_, while Fat_ox_ only showed a small difference at 4 versus 6 min (~0.01 g/min; < 2% of absolute Fat_ox_). Consequently, although shorter averaging windows introduce some variability, these differences are unlikely to have a meaningful impact on practical assessments of pulmonary gas exchange and substrate utilization. There were no differences for V̇O_2_, V̇CO_2_, or Fat_ox_ across averaging windows during cycling, and as such, and comparable to the running data, the practical relevance of stage duration and averaging method manipulations was limited. This lack of differences in cycling and the minimal effect sizes observed in running fall within the instrumental measurement error [38]. No difference was observed between 30‐ and 60‐s measurement analysis for each averaging window for either cycling or running.

### 4.2 Exercise Intensity and Modality

When findings were analyzed based on exercise intensity (moderate vs. heavy), no V̇O_2_ differences were observed during running, while V̇CO_2_ was lower at 3 vs. 6 min at moderate intensity (−18 mL/min) and at 3 versus 4, 5, and 6 min at heavy intensity (−39 mL/min vs. 6 min). There were no differences for Fat_ox_ between moderate and heavy intensity, suggesting that a 3 min stage duration may accurately estimate fat oxidation and maximal fat oxidation. In cycling, significant differences in V̇O_2_ and V̇CO_2_ were observed between moderate versus heavy intensity at 3 min (*p* = 0.001) and 4 min (*p* = 0.045) suggesting that V̇O_2_ kinetics differ at 3 and 4 min depending on exercise intensity. However, these differences did not result in lower V̇O_2_ values earlier in the stage. The intensity effect, which appeared to be more pronounced in running than in cycling, does not align with previous literature [39, 40, 41], which typically reports a greater V̇O_2_ slow component in cycling compared to running (or even its non‐existence in running; [39]). A few mechanisms could explain this difference in results between cycling and running. These include a break observed between each running stage (i.e., 30 s for BLa sampling), which may have limited fatigue development and potentially the development of the V̇O_2_ slow component [42]. Importantly, when comparing participants performing both running and cycling incremental tests (*n* = 23), no averaging window × exercise modality interaction or main effect of exercise modality were detected on V̇O_2_ and V̇CO_2_ regardless of the exercise intensity. This within‐participant analysis provides a more rigorous and direct comparison between exercise modalities, although the relatively small sample size may limit its direct extrapolation to the overall cohort of athletes in this study. The findings from both running and cycling modality are likely the result of the influence of the slow component that develops during heavy exercise [18] where V̇O_2_ continues to increase for several minutes before reaching steady state [43]. Absolute V̇O_2_ and V̇CO_2_ differences between 3 and 6 min remained small for both intensities and exercise modalities, suggesting that a 3 min step duration could be enough to assess respiratory gases in most of the situations.

### 4.3 Fitness Status

Another factor that may impact the effect of stage duration during an incremental steps test is the fitness status of participants, as trained individuals typically present faster V̇O_2_ kinetics (i.e., shorter time to reach a steady state), regardless of the exercise modality [27]. In this study both running and cycling displayed similar V̇O_2_, V̇CO_2_ and Fat_ox_ between athletes of “excellent” and “good or below” fitness level. While some participants were classified as “good or below” fitness status, their V̇O_2_peak (~47 mL/kg/min) likely minimized differences with athletes of “excellent” fitness status, as they were all physically active, healthy individuals. The lack of a “fitness level” effect in this study encourages researchers/practitioners to adopt shorter stage durations to reduce the overall test duration, limit the influence of the V̇O_2_ slow component and metabolic drift, and minimize fatigue that could compromise maximal performance in later stages [6, 7]; or increase the number of stages that can be completed prior to exhaustion (or a specific cut‐off reached) through a reduction in speed/power increments between stages, improving the accuracy of thresholds determination. Therefore, a 3 or 4 min step duration is suggested to accurately assess V̇O_2_, V̇CO_2_, and Fat_ox_ independently of the individual fitness status.

### 4.4 Biological Sex

When the influence of biological sex was analyzed, an effect of averaging windows on V̇O_2_ during running was observed in males, with lower values for shorter durations, but not in females. Notably, the Δ between 3 and 6 min for V̇O_2_ was larger in males (−33 mL/min) than in females (−15 mL/min). These sex‐specific differences may be partly explained by the differing incremental running speed between stages in running, which were slightly larger for males (+0.7 km/h on average) than for females (+0.6 km/h). Despite these observations, no difference in V̇CO_2_ and Fat_ox_ between sexes was observed in running. Similarly, no averaging window difference, regardless of the variable, was observed in cycling, suggesting that previous recommendations could be followed regardless of biological sex.

### 4.5 Age

Although previous studies reported slower V̇O_2_ kinetics in older individuals [28], no differences in averaging window for V̇O_2_ and V̇CO_2_ were observed in this study, regardless of exercise modality. This lack of difference may be due to a smaller age gap than previous studies (e.g., 25 vs. 68 years; [28]), as here we compared senior (30 ± 6 years for cycling; 31 ± 5 years for running) and master (51 ± 8 years for cycling; 48 ± 6 years for running) individuals, with an average age difference of 19 years. Furthermore, a slower V̇O_2_ kinetics appear to be more strongly linked to a fitness status declines with age, rather than aging itself [44, 45]. It is worth noting that the participants' V̇O_2_peak (i.e., fitness status) in the current study did not differ substantially between seniors and masters (~58 [senior] vs. ~52 mL/min/kg [masters]), which could partially explain the absence of an age effect.

## 5 Limitations

This study is not without limitations. The effect of the averaging window was analyzed as a proxy of stage duration during a single incremental test; therefore, the 6 min stage duration may have elicited a carryover effect on subsequent stages, which were then analyzed for differences in the averaging window. However, differences between a 3 and 6 min averaging window for V̇O_2_ and V̇CO_2_ were found to constantly be < 1.2% for each condition analyzed, which is similar to or smaller than the between‐day reliability of these variables even when diet and exercise are standardized [12, 13, 46]. The current within‐stage analysis from a single incremental test removes variability caused by differences in nutritional status, time of day, and other external factors typically associated with trials conducted across separate visits. Future research could confirm these findings using assessments with different stage durations across separate trials. Similarly, for the within‐participant comparison of exercise modality, the analysis was performed as an intra‐stage comparison (i.e., only comparisons of different averaging window within the same stage), limiting the influence of external factors. Furthermore, although exercise modalities were compared at moderate and heavy domains, intensity was not matched as a percentage of metabolic thresholds. Also, differences in muscle mass and perhaps sport specialism could influence absolute V̇O_2_ measured. However, similar responses were found for averaging window main effects and interactions (*p* = 0.13–0.94) with a very small effect size (*η*_p_^2^ = 0.00–0.02), suggesting that similar outcomes are likely to be found when exercise intensity closely aligns between exercise modalities. Finally, the differences in increments of power/speed during the step test were not standardized between participants, with a potential impact on the step increase relative to the individual maximal capacity. The increments were set based on the participant characteristics to undertake 7–9 stages before reaching a RER > 1, and the average number of stages (~8) did not differ between exercise modalities, suggesting that most participants experienced a similar relative intensity increase. It should also be noted that V̇O_2_ kinetics parameters (e.g., time constants) were not directly assessed in this study; therefore, discussion related to V̇O_2_ kinetics should be interpreted cautiously.

## 6 Perspectives

The results of this study support and extend earlier findings by Weltman et al. [8], showing minimal physiological differences between 3 and 10 min stages during treadmill testing. The results of this study indicate that V̇O_2_ and V̇CO_2_ display very small differences when separate averaging windows from a single incremental step test—sampled for 30 s at 3, 4, 5, and 6 min—were compared, with a 3 versus 6 min difference typically < 1% (< 30 mL/min) than the absolute value sampled for both running and cycling exercise. The averaging window was not affected by exercise intensity or modality, fitness status, and age, while its effect was ~50% smaller in females than males (e.g., 33 vs. 15 mL/min Δ between 3 and 6 min). Despite the small differences in V̇O_2_ and V̇CO_2_ for separate averaging windows, Fat_ox_ did not meaningfully differ between any of the conditions analyzed, with a maximum mean difference of 0.02 g/min, indicating that stages as short as 3 min can be used to accurately assess Fat_ox_ and derivate measures such as maximal fat oxidation. These results suggest that a 3 min stage duration could be used for V̇O_2_, V̇CO_2_, and Fat_ox_ assessment, as previously recommended [47], which would reduce the total duration of the exercise and limit fatigue development, as longer durations may be impacted by an underdeveloped “durability” [48, 49], especially in lesser trained athletes [50, 51]. A shorter stage duration could also be beneficial to reduce the rate of speed/power increments between stages or increase the number of stages performed prior to exhaustion (or a specific cut‐off reached), subsequently enhancing the accuracy of metabolic thresholds detection. These results provide contemporary validation for the use of shorter stage durations in exercise testing, supporting the rationale behind 3 min stages that have been widely adopted internationally over the last ~30 years.

## 7 Conclusion

In a large cohort of trained individuals, an incremental step test comprising 3 min stages enables accurate assessments of V̇O_2_, V̇CO_2_, and Fat_ox_ in both cycling and running, despite a break for capillary blood sampling during running. Results from a 3 min time window do not meaningfully differ from a 6 min stage duration, and can be used to minimize fatigue and allow smaller speed/power increments to enhance the accuracy of metabolic thresholds detection. A 3 min stage duration is appropriate irrespective of exercise intensity, fitness status, age, and biological sex in athletic cohorts.

## Ethics Statement

The laboratory protocol received ethical approval from the Loughborough University Ethics Reviews Sub‐Committee (Project ID G13‐P1).

## Consent

The authors have nothing to report.

## Conflicts of Interest

The authors declare no conflicts of interest.

## Supporting information


Data S1.

## Data Availability

The raw data are provided in the Supporting Information. Additional data are available from the corresponding author upon reasonable request.
