# Supplementary material for: Factors Affecting V̇O2 and Fat Oxidation Responses During Step Incremental Exercise
Source: Scand J Med Sci Sports. 2025 Aug 1;35(8):e70110. doi: 10.1111/sms.70110 (PMC12317159; doi:10.1111/sms.70110)
Supplement: Supplementary file 1 — Data S1. [file SMS-35-e70110-s001.docx]

**Supplementary materials**

**Factors affecting V̇O₂ and fat oxidation responses during step incremental exercise.**

Mougin, L. ^1*^, Bailey, S.J. ^1^, Burnley, M. ^1^, Pearce, R. ^2^, Mears, S.A.^1^, Zanini, M. ^1,3^

^1^School of Sport, Exercise and Health Sciences, Loughborough University, Loughborough, United Kingdom.

^2^Loughborough Sport, Loughborough University, Loughborough, United Kingdom.

^3^School of Education, Childhood, Youth and Sport, The Open University, Milton Keynes, United Kingdom.

*****Corresponding author**

Loïs Mougin, School of Sport, Exercise and Health Sciences, Loughborough University, Loughborough, Leicestershire LE11 3TU, UK

Email: [lois.mougin@gmail.com](mailto:lois.mougin@gmail.com) - [L.m.b.h.mougin@lboro.ac.uk](mailto:L.m.b.h.mougin@lboro.ac.uk); Phone: (+33) 789516446.

***Supplementary table 1.*** Differences in averaging V̇O₂, V̇CO₂, and Fat_ox_ over 30 or 60 s after 3, 4, 5 compared to 6 minutes (average on the last 30 s) for each stage performed in in running and cycling.

|  | Exercise  modality | Condition | Δ from 6 min | | |
| --- | --- | --- | --- | --- | --- |
|  |  |  | 3 min | 4 min | 5 min |
| V̇O₂ (mL/min) | Running | 30 s | -28 ± 73 | -15 ± 71 | -8 ± 69 |
|  |  | 60 s | -29 ± 70 | -21 ± 67 | -11 ± 61 |
|  | Cycling | 30 s | -2 ± 94 | 4 ± 80 | 5 ± 78 |
|  |  | 60 s | -3 ± 85 | 0 ± 76 | 2 ± 69 |
| V̇CO₂ (mL/min) | Running | 30 s | -34 ± 88 | -12 ± 82 | -6 ± 79 |
|  |  | 60 s | -40 ± 86 | -23 ± 78 | -9 ± 73 |
|  | Cycling | 30 s | 20 ± 107 | 20 ± 101 | 16 ± 96 |
|  |  | 60 s | 12 ± 100 | 15 ± 97 | 15 ± 86 |
| Fat_ox_ (g/min) | Running | 30 s | -0.00 ± 0.11 | 0.01 ± 0.10 | 0.00 ± 0.09 |
|  |  | 60 s | -0.00 ± 0.11 | 0.01 ± 0.09 | 0.00 ± 0.08 |
|  | Cycling | 30 s | 0.00 ± 0.11 | 0.01 ± 0.11 | 0.01 ± 0.10 |
|  |  | 60 s | 0.00 ± 0.11 | 0.00 ± 0.10 | 0.01 ± 0.09 |

Mean ± SD. V̇O₂: Oxygen uptake; V̇CO₂: Carbon dioxide production; Fat_ox_: Fat oxidation

***Supplementary table 2.*** Differences in measuring Fat_ox_ after 3, 4, 5 compared to 6 minutes for each stage depending on intensity, fitness status, age and biological sex, in running and cycling.

|  |  |  | Δ Fat_ox_ from 6 min (g/min) | | | Main effect | Interaction |
| --- | --- | --- | --- | --- | --- | --- | --- |
|  |  |  | 3 min | 4 min | 5 min |  |  |
| Intensity | Running | Moderate | -0.00 ± 0.11 | 0.01 ± 0.11 | -0.01 ± 0.10 | *P* = 0.288  F = 1.1  η_p_^2^ = 0.00 | *P* = 0.114  F = 2.0  η_p_^2^ = 0.00 |
|  |  | Heavy | 0.01 ± 0.10 | 0.01 ± 0.09 | 0.01 ± 0.09 |  |  |
|  | Cycling | Moderate | 0.00 ± 0.11 | -0.00 ± 0.11 | 0.01 ± 0.10 | *P* = 0.864  F = 0.0  η_p_^2^ = 0.00 | *P* = 0.080  F = 2.3  η_p_^2^ = 0.01 |
|  |  | Heavy | 0.00 ± 0.11 | 0.01 ± 0.10 | -0.00 ± 0.10 |  |  |
| Fitness status | Running | Excellent | 0.00 ± 0.11 | 0.01 ± 0.10 | 0.01 ± 0.10 | *P* = 0.195  F = 1.7  η_p_^2^ = 0.00 | *P* = 0.641  F = 0.6  η_p_^2^ = 0.00 |
|  |  | Good | -0.01 ± 0.11 | 0.01 ± 0.11 | -0.00 ± 0.18 |  |  |
|  | Cycling | Excellent | -0.01 ± 0.11 | -0.00 ± 0.11 | 0.01 ± 0.10 | ***P* = 0.041**  F = 4.2  η_p_^2^ = 0.01 | ***P* = 0.023**  F = 3.2  η_p_^2^ = 0.01 |
|  |  | Good | 0.02 ± 0.11 | 0.02 ± 0.11 | 0.01 ± 0.10 |  |  |
| Age | Running | < 40 yr | 0.01 ± 0.12 | 0.02 ± 0.11 | 0.01 ± 0.09 | ***P* = 0.020**  F = 5.5  η_p_^2^ = 0.01 | ***P* = 0.044**  F = 2.7  η_p_^2^ = 0.00 |
|  |  | ≥ 40 yr | -0.01 ± 0.10 | 0.00 ± 0.09 | -0.00 ± 0.09 |  |  |
|  | Cycling | < 40 yr | -0.00 ± 0.10 | 0.01 ± 0.09 | 0.01 ± 0.08 | *P* = 0.670  F = 0.2  η_p_^2^ = 0.00 | *P* = 0.601  F = 0.6  η_p_^2^ = 0.00 |
|  |  | ≥ 40 yr | 0.01 ± 0.12 | 0.00 ± 0.12 | 0.01 ± 0.11 |  |  |
| Biological sex | Running | Females | -0.00 ± 0.09 | 0.00 ± 0.08 | 0.00 ± 0.07 | *P* = 0.370  F = 0.8  η_p_^2^ = 0.00 | *P* = 0.757  F = 0.4  η_p_^2^ = 0.00 |
|  |  | Males | 0.00 ± 0.12 | 0.01 ± 0.11 | 0.01 ± 0.10 |  |  |
|  | Cycling | Females | -0.02 ± 0.09 | -0.01 ± 0.08 | -0.01 ± 0.07 | ***P* = 0.032**  F = 4.6  η_p_^2^ = 0.01 | *P* = 0.152  F = 1.8  η_p_^2^ = 0.00 |
|  |  | Males | 0.01 ± 0.12 | 0.01 ± 0.11 | 0.01 ± 0.11 |  |  |

Mean ± SD. V̇CO₂: Carbon dioxide production. η_p_^2^: partial-eta squared (small (0.01 - 0.06), medium (0.06 - 0.14), and large (> 0.14)).

***Supplementary table 3.*** Differences in measuring V̇CO₂ after 3, 4, 5 compared to 6 minutes for each stage depending on intensity, fitness status, age and biological sex, in running and cycling.

|  |  |  | Δ V̇CO₂ from 6 min (mL/min) | | | Main effect | Interaction |
| --- | --- | --- | --- | --- | --- | --- | --- |
|  |  |  | 3 min | 4 min | 5 min |  |  |
|  | Running |  | -34 ± 88  ^$$$ &&& ###^ | -12 ± 82  ^###^ | -6 ± 79 | *P* < 0.001  F = 37.1  η_p_^2^ = 0.06 | ***-*** |
|  | Cycling |  | 20 ± 107  ^###^ | 20 ± 101  ^###^ | 16 ± 96  ^##^ | *P* < 0.001  F = 8.3  η_p_^2^ = 0.02 | ***-*** |
| Intensity | Running | Moderate | -18 ± 87  ^#^ | -3 ± 90 | -8 ± 83 | *P* = 0.086  F = 3.0  η_p_^2^ = 0.01 | ***P* = 0.011**  F = 3.7  η_p_^2^ = 0.01 |
|  |  | Heavy | -39 ± 75  ^$ &&& ###^ | -18 ± 71  ^#^ | -6 ± 77 |  |  |
|  | Cycling | Moderate | 38 ± 108  ^###^ | 37 ± 97  ^###^ | 25 ± 92  ^##^ | ***P* = 0.004**  F = 8.5  η_p_^2^ = 0.03 | ***P* = 0.026**  F = 3.1  η_p_^2^ = 0.01 |
|  |  | Heavy | 11 ± 106 | 8 ± 99 | 2 ± 109 |  |  |
| Fitness status | Running | Excellent | -28 ± 94 | -11 ± 86 | -6 ± 86 | *P* = 0.323  F = 1.0  η_p_^2^ = 0.00 | *P* = 0.090  F = 2.2  η_p_^2^ = 0.00 |
|  |  | Good | -44 ± 76 | -15 ± 77 | -5 ± 66 |  |  |
|  | Cycling | Excellent | 14 ± 109 | 11 ± 98 | 11 ± 96 | *P* = 0.074  F = 3.2  η_p_^2^ = 0.01 | *P* = 0.237  F = 1.4  η_p_^2^ = 0.00 |
|  |  | Good | 27 ± 105 | 30 ± 103 | 22 ± 95 |  |  |
| Age | Running | < 40 yr | -32 ± 87 | -15 ± 87 | -9 ± 86 | *P* = 0.295  F = 1.1  η_p_^2^ = 0.00 | *P* = 0.725  F = 0.4  η_p_^2^ = 0.00 |
|  |  | ≥ 40 yr | -36 ± 90 | -9 ± 76 | -2 ± 71 |  |  |
|  | Cycling | < 40 yr | 11 ± 103 | 13 ± 85 | 17 ± 81 | *P* = 0.372  F = 0.8  η_p_^2^ = 0.00 | *P* = 0.320  F = 1.2  η_p_^2^ = 0.00 |
|  |  | ≥ 40 yr | 25 ± 110 | 23 ± 109 | 15 ± 104 |  |  |
| Biological sex | Running | Females | -35 ± 76 | -12 ± 77 | -4 ± 64 | *P* = 0.891  F = 0.0  η_p_^2^ = 0.00 | *P* = 0.975  F = 0.1  η_p_^2^ = 0.00 |
|  |  | Males | -34 ± 92 | -13 ± 85 | -6 ± 84 |  |  |
|  | Cycling | Females | 4 ± 87 | 6 ± 81 | 9 ± 73 | *P* = 0.124  F = 2.4  η_p_^2^ = 0.01 | *P* = 0.343  F = 1.1  η_p_^2^ = 0.00 |
|  |  | Males | 24 ± 111 | 23 ± 105 | 18 ± 101 |  |  |

Mean ± SD. V̇CO₂: Carbon dioxide production. η_p_^2^: partial-eta squared (small (0.01 - 0.06), medium (0.06 - 0.14), and large (> 0.14)). #: different to 6 min. &: different to 5 min. $: different to 4 min. 3 symbols for P < 0.001; 2 symbols for P < 0.01; 1 symbol for P < 0.05.
